# Management algorithm for alcohol withdrawal syndrome, alcohol dependence (AD) and AD with anxiety in the Indian population: a narrative review with expert opinion

**DOI:** 10.3389/fpsyt.2026.1758995

**Published:** 2026-03-13

**Authors:** M. Suresh Kumar, Mrugesh Vaishnav, Gautam Saha, Deepak Raheja

**Affiliations:** 1 Psymed Hospital, Chennai, Tamil Nadu, India; 2 Samvedana Happiness Hospital, Ahmedabad, Gujarat, India; 3 Clinic Brain, Neuropsychiatric Institute and Research Center, Barasat, Kolkata, India; 4 Hope Care India, Psychiatric & Rehab Centre, New Delhi, India

**Keywords:** alcohol dependence, alcohol withdrawal syndrome, anxiety, benzodiazepines, physicians, psychiatrists

## Abstract

Alcohol dependence (AD) is associated with several physical and psychiatric implications such as those observed in alcohol withdrawal syndrome (AWS) and AD with co-morbid anxiety. With the increasing burden of AD in India, both physicians and psychiatrists face unique challenges while managing patients with AWS, AD, and AD with anxiety. Physicians are often the first point of contact for patients presenting with alcohol use-related medical complications but lack clear management directives that distinguish their role from that of psychiatrists. To address this need gap, a series of five focus group discussions were conducted with a total of 57 expert physicians and psychiatrists. The expert opinions are presented along with supporting evidence from current literature based on a comprehensive literature review. This practice and policy review presents guidance for physicians and psychiatrists on the optimal screening, diagnosis, and treatment pathways to follow for patients presenting with each of the three indications. It highlights the importance of accurately diagnosing and distinguishing the three indications by using distinct inventories of presenting symptoms, questions to be asked as a part of comprehensive history-taking of the patient, screening questionnaires and tools, and laboratory investigations. It also discusses the roles of different classes of drugs in addressing the diverse presentations and comorbidities associated with each indication. Benzodiazepines, especially long-acting ones like chlordiazepoxide, remain the mainstay for detoxification protocols for both AWS and AD. Physicians need to work in conjunction with psychiatrists and refer the patient when required, based on distinct criteria such as severity of psychiatric comorbidities, complications of withdrawal, and behavioral issues leading to persistent alcohol use and relapses. The application of this holistic management approach that encompasses the multifaceted nature of alcohol-use related disorders will help reduce relapse rates and ensure optimal patient outcomes.

## 1 Introduction

Alcohol dependence (AD) has a multifaceted nature with a broad range of physical, mental, and social implications. Out of the approximately two billion global users of alcohol, 76.3 million suffer from at least one disorder due to their alcohol use (1). In India, alcohol abuse is a significant concern, with alcohol use-related disorders or comorbidities reported to account for 0.26 million alcohol-attributable deaths almost a decade ago in 2016 (2). These numbers are presumably under-reported in clinical practice and have increased substantially since then, as a considerable proportion of alcohol consumption remains unrecorded (3).

AD-related disorders can be physical, such as hepatic cirrhosis, neoplasia, pancreatitis; psychiatric, such as anxiety, depression, personality disorders; or a combination of both, such as alcohol withdrawal syndrome (AWS) that develops after sudden reduction or cessation of alcohol consumption. These conditions are independently associated with a poor prognosis and an increase in morbidity and mortality for the patients (1, 4, 5). Frequent relapses further add complexities to treatment efforts for patients with AD (5). Treatment with appropriate therapy also remains challenging due to the complexity and variability of the condition itself and other variables such as coexisting disorders, stage of addiction, pattern of drinking, treatment regimen and compliance, and genotype (5). AD with co-existing anxiety is highly prevalent in clinical practice and requires specialized approaches. But clinical evidence for its diagnosis and treatment remains limited (6). Thus, there is a need for a holistic management approach for AD-related disorders that encompasses all the factors for consideration, stabilize the patients, and reduces relapse rates and the need for further courses of treatment (7).

The role of physicians in preventing and treating substance use disorders (SUDs) is significant in scale (8). In India, physicians are the first point of contact for AD-related medical complications and thus well-positioned to offer evidence-based treatment to the patients. However, the critical role of physicians may be limited by the lack of adequate training in evaluating and managing AD-related cases (9, 10). This resonates with the current lack of management algorithms specifically for physicians in Indian clinical practice.

This policy and practice review, supported by expert opinion, aims to guide physicians and psychiatrists on the optimal management pathways to follow for patients presenting with alcohol use-related disorders—AWS, AD, and AD with anxiety. The review will also discuss the alcohol use-related challenges specific to the Indian scenario and delineate the integrated and multidisciplinary approaches to tackle them.

## 2 Methods

A series of five focus group meetings was conducted between November 2024 and January 2025 with 40 psychiatrists and 14 physicians in total from across India. The participants were selected using purposeful sampling based on geographical location, clinical experience (minimum 10 years of clinical practice), and availability to attend the meeting. Physicians were required to have clinical experience in managing AD and AWS cases on a regular basis. The first four regional meetings were conducted with experts, including the authors, representing the four major geographical regions in India (North, South, East and West; **Supplementary Table 1**). In each regional meeting, a slide deck was presented with panel questions and supporting clinical evidence for discussion, based on a pre-specified agenda (**Supplementary Table 2**). The experts discussed the epidemiology of cases in their clinical practice and challenges in management. The discussion mainly focused on 1) screening and diagnosis (clinical history, presenting symptoms, scales or questionnaires, physical/mental/laboratory examination), 2) treatment modalities, 3) role of physicians and when to consult a psychiatrist, for each of the three indications—AWS, AD, and AD with anxiety. The authors used the findings from the four regional meetings in conjunction with the existing guidelines to formulate the screening, diagnosis and treatment algorithms for the three indications. The authors then presented the algorithms in a fifth focus group meeting, attended by the representatives from the previous four meetings. The inputs from the fifth meeting were incorporated into the algorithms and finalized after approval from all the panel members.

In this narrative review, the experts’ opinions are presented along with supporting evidence from the literature. The research questions were pre-specified based on the agenda of the focus group meetings (**Supplementary Table 2**). The search strategy included a literature search of the major relevant databases (PubMed, Scopus, Cochrane Library, Google Scholar) using search strings based on the keywords— ‘alcohol withdrawal syndrome’, ‘alcohol dependence’, ‘anxiety’, ‘benzodiazepines’, ‘physicians’, ‘psychiatrists’. The guidelines for alcohol addiction were searched manually on the official websites of the American Society of Addiction Medicine (ASAM), National Health Mission, National Institute for Health and Care Excellence (NICE), and Google (https://www.google.com/). No filters were applied to specify the type of article or the year of publication. Citations within the articles were also explored, if they appeared relevant to the current review. The final reference list was selected by the authors after assessing the relevance of the study title and abstract to the topics covered in this review.

## 3 Epidemiology

Alcohol is one of the most abused substances in India (3). There are several studies estimating the burden of alcohol use in India, but there is a paucity of data available on the prevalence of AD and AWS in Indian clinical practice, possibly due to misdiagnosis, lack of awareness, and disparity in treatment (10). The available studies do not differentiate between the number of cases encountered by psychiatrists and physicians (11).

According to National Family Health Survey-4 (n= 103,411 males and 699,686 females), most people consuming alcohol (male and female) were in the age group of 40–49 years (36.7%) and 25–39 years (35.6%) and the least in the 15–24-year age group (16.1%). In India, heavy drinking starts in people aged 15–24 years (12). Overall, alcohol consumption is predominantly observed in males (29.2%) compared to females (1.2%), but with more adverse effects in females (13, 14). A community cohort study reported that 15% of people who drank alcohol casually developed alcohol use disorder (AUD) within six years (15).

In an exploratory study (n=40) in India with a purposive sampling technique, 42.5% of the patients developed AD between 21 and 30 years of age (16). In a survey-based study, 62% of the Indian intensive care unit doctors (N = 211) reported that AUD cases constituted 10% of the total cases (10). Patients >60 years of age with AD or AWS are not commonly encountered in clinical practice but may report a more severe or prolonged withdrawal due to comorbidities like hypertension, diabetes, coexisting somatic diseases, and electrolyte disturbances (17). Almost all the individuals visiting the outpatient department for treatment of AUD are males; social barriers prevent women from seeking treatment (18).

AD with anxiety is commonly encountered among Indian patients. In a single-centered, cross-sectional study (n=90) on patients with AD, 37.7% had mild, 46.7% had mild-moderate, and 15.6% had moderate-severe anxiety symptoms as measured by the Hamilton rating scale for anxiety (HAM-A) score. The anxiety symptoms were shown to correlate significantly with the duration of alcohol consumption (19). Anxiety, the most prevalent psychiatric comorbidity with AUD, is associated with a more severe illness, higher disability, a tendency towards heavy drinking, worse treatment outcomes, and a greater number of hospitalizations than those with AUD alone (20).

### 3.1 Expert opinion

Both physicians and psychiatrists agreed with the increased alcohol use in recent times across urban and semi-urban areas due to westernization and changing societal norms. The normalization of casual drinking and exposure to alcohol-related content on social media has also led to increased alcohol use in women and the early age of initiation in young adults. Many patients self-medicate alcohol to treat anxiety and may experience anxiety on depletion of alcohol levels in their system, thus creating a complex cycle. **Table 1** and **Supplementary Table 3** list the epidemiology of cases encountered by physicians and psychiatrists in clinical practice. The physicians encountered fewer numbers of weekly AD and AWS patients compared to psychiatrists. However, the age and sex distributions of the patients were similar for both practitioners. Most of the patients across the three indications were in the 40–60 years age group and males (**Table 1**).

**Table 1:** Alcohol use-related cases in clinical practice in India.

Demographics	No. of cases encountered by physicians	No. of cases encountered by psychiatrists
1) Indication		
AD	8–15 patients/week	50–60 patients/week
AWS	5–10 patients/week	30–40 patients/week
AD with anxiety	20–60 patients/week	–
2) Age		
<20 years of age	5%–10%	5%–10%
20–40 years	50%–70%	50%–70%
40–60 years	10%–20%	10%–20%
>60 years	1%–5%	1%–5%
3) Gender		
• Male	95%–99%	95%–99%
• Female	1%–5%	1%–5%

AD, alcohol dependence; AWS, alcohol withdrawal syndrome.

## 4 Challenges in the management of AD and its manifestations in India

Several barriers at both the patient and physician levels can hinder the management of AD (**Table 2**). In India, the treatment gap for AUDs is considerable as there is a lack of routine screening for alcohol use and its potential complications (15). The alcohol dependence treatments are primarily based in tertiary centers and focus on the treatment of complications of AD but not their prevention. Relapse rate can range up to 90% across treatment centers in India (21). Lack of motivation towards abstinence is one of the major barriers in treatment for AD. Lack of family support, stigma around the treatment, low socioeconomic status, and financial constraints further contribute to poor patient outcomes. Psychiatric comorbidities and certain personality traits, such as narcissism and anti-social tendencies, also affect the course of recovery for a patient with AD (22) (**Table 2**). Thus, adoption of individualized approaches and scaling up of evidence-based interventions is needed for the treatment of AD (12).

**Table 2:** Barriers challenging the management of AUDs (22, 26, 27).

Barriers at the patient level	Barriers at the physician level
• Not acknowledging AUD as a concern • Not reporting alcohol use accurately • Concerns for privacy • Lack of insurance • Lack of available treatment facilities • Lack of referral • Lack of motivation • Lack of family support • Stigma around the treatment • Low socio-economic status • Financial constraints	• Not screening/identifying AUD and its implications • Not identifying symptoms of ‘at-risk’ patients • Not taking detailed patient history • Lack of training resulting in inadequate treatment • Sub-optimal dose used • Misdiagnosis • Treatment for secondary complications over AUD • Not using validated screening tools • Lack of integration between different specialists (gastroenterologists, psychiatrists, etc.) • Dearth of social workers or clinical psychologists

AUD, alcohol use disorder.

The treatment strategies in India focus predominantly on patients in acute need as opposed to preventative measures for those at risk of developing alcohol-related complications in the future (23). Psychiatrists practicing in India have reported a dearth of social workers or clinical psychologists for referral of patients following treatment for acute presentation (23) (**Table 2**). Lack of trained physicians also contributes to the highest reported treatment gap (86.3%) for AUDs compared to other mental health disorders (24, 25).

### 4.1 Expert opinion

**Figure 1:**
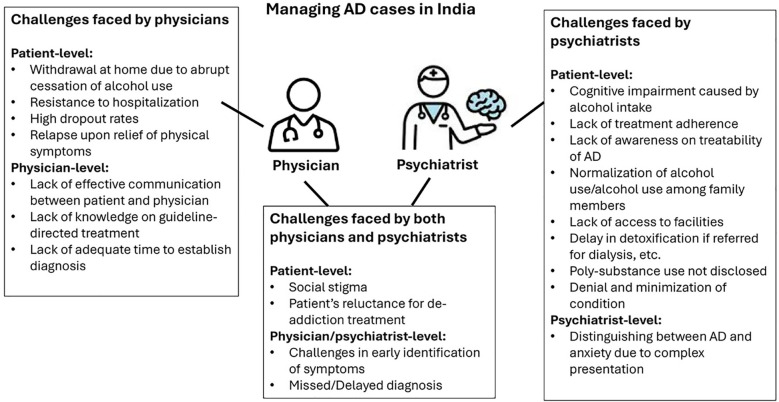
Unique and common challenges faced by physicians and psychiatrists while managing AD cases in India. AD, alcohol dependence.

Apart from the challenges identified in published literature, there are certain unique challenges faced by physicians and psychiatrists in their clinical practice in India (**Figure 1**). Missed or delayed diagnosis remains one of the primary concerns while managing AD and its associated disorders. Accurate diagnosis of AD with anxiety is frequently missed, leading to treatment of anxiety alone, without consideration of underlying AD. Sleep-related disorders are often not recognized as possible withdrawal symptoms. Comprehensive patient history is critical but overlooked due to time and personnel constraints. Missing early warning signs like daily solitary drinking that can develop into AD over 10–15 years results in a significantly delayed diagnosis. Patients may also present directly with severe dependency or secondary complications like liver damage, cardiovascular disorders, or depression.

## 5 Screening and diagnosis: AWS, AD, and AD with anxiety

The accurate and distinct diagnosis of AWS, AD, and AD with anxiety is clinically important. AWS can be associated with complicated withdrawal, like delirium tremens (DT) or hallucinosis, that can be fatal if untreated (28). A study on Indian patients has reported a mortality rate of 20% in patients with untreated complicated withdrawal (28). AD can rapidly progress to withdrawal if left undiagnosed and untreated (29). Anxiety underlying AD can worsen the symptoms of AD and lead to poor patient outcomes, especially if not recognized (29, 30). Thus, there is a need for a distinct approach to screen and diagnose each of the three indications.

The guidelines for the Ministry of Health & Family Welfare, Government of India, highlight the role of detailed case history from every patient due to the possibility of a significant lag between AD development and patients seeking treatment for withdrawal (31). Physical examination to assess the features of AWS and mental state examination (MSE) to identify complicated withdrawal should be performed along with laboratory investigations before initiating the course of treatment (31). The American Society of Addiction Medicine (ASAM) also recommends gaining information from collateral sources such as family and friends for a detailed case history to determine if the patient is experiencing or is at risk for developing AWS (32). Co-occurring psychiatric disorders and SUDs should not be ruled out while examining patients with AWS (32).

CIWA-Ar is a validated 10-item assessment tool and the gold standard for quantifying the severity of AWS and guiding the treatment approach for AWS (**Supplementary Table 4**) (33, 34). Implementation of a CIWA-Ar-based alcohol withdrawal protocol with targeted training significantly improves quality of care, patient safety, and treatment effectiveness in a general medical/surgical hospital setting (35). CIWA-Ar scale is also extensively used in Indian clinical settings and has been proven to be valid and with high internal consistency among the items when assessing patients with AWS (36, 37).

Several screening and assessment tools have been extensively studied and validated in the Indian population for AD and AD with anxiety (**Table 3**). The Diagnostic and Statistical Manual-5 lists down signs and symptoms to be used as diagnostic criteria for alcohol withdrawal and AUD (38) and has been used in the Indian population for diagnosing AUD (39, 40). The Severity of Alcohol Dependence Questionnaire (SADQ) is a short, self-administered questionnaire with ‘Physical Withdrawal’, ‘Affective Withdrawal’, ‘Withdrawal Relief Drinking’, ‘Alcohol Consumption’, and ‘Rapidity of Reinstatement’ subscales to assess the severity of AD (41). Developed by World Health Organization (WHO), Alcohol Use Disorders Identification Test (AUDIT) is a 10-item validated questionnaire widely used in India and worldwide for brief assessment of hazardous drinking patterns (42, 43). AUDIT scores have shown a significant correlation between the severity of AD and lifestyle alcohol consumption in Indian patients with AD (**Table 3**) (44). Alcohol, Smoking and Substance Involvement Screening Test (ASSIST), also developed by WHO, spans the use of all psychoactive substances, including alcohol and associated problems over the preceding three-month period. ASSIST and its linked brief interventions have been used in more than one Indian workplace settings (45, 46). Cutting down, Annoyance by criticism, Guilty feeling, and Eye-openers (CAGE) consists of four yes/no items, can be administered in 30 seconds and is easy to memorize (47, 48). CAGE has been used in several studies to screen for alcohol misuse in Indian population (**Table 3**) (49, 50).

**Table 3:** Commonly used tools for screening and severity assessment in patients with AWS, AD, and AD with anxiety (33, 38–54).

Indication	Tool/Questionnaire	Utility	Studied/Validated in Indian population
AWS	CIWA-Ar	Quantifying severity of AWS	Yes
AD	DSM-5	Quantifying severity of AD	Yes
	CAGE	Detection of alcohol abuse and dependence	Yes
	AUDIT	Assessing the severity of AD	Yes
	ASSIST	Identification of poly substance use with alcohol	Yes
	SADQ	Assessing the severity of AD	Yes
AD with anxiety	GAD-7	Screening and assessing the severity of anxiety	Yes
	HAM-A	Assessing the severity of anxiety	Yes

AWS, alcohol withdrawal syndrome; AD, alcohol dependence; CIWA-Ar, Clinical Institute Withdrawal Assessment for Alcohol revised; DSM-5, Diagnostic and Statistical Manual, 5th Edition; AUDIT, Alcohol Use Disorders Identification Test; ASSIST, Alcohol, Smoking and Substance Involvement Screening Test; SADQ, Severity of Alcohol Dependence Questionnaire; HAM-A, Hamilton Anxiety Rating Scale; GAD-7, Generalized anxiety disorder 7-item.

The Generalized Anxiety Disorder 7-item (GAD-7) scale is a 4-point Likert-scaled item, used as a screening tool as well as to assess the symptom severity for different types of anxiety (51, 52). It has shown comparable psychometric properties between the rural Indian population and Western settings, supporting its use in Indian clinical practice (**Table 3**) (51). Hamilton Anxiety Rating Scale (HAM-A), a clinician-based questionnaire, consists of 14 symptom-defined elements to measure the severity of anxiety symptoms, and spans both psychological and somatic symptoms. One of the oldest and most widely used scales, it has also been used to demonstrate the high prevalence of anxiety among Indian patients with AUD (**Table 3**) (53, 54). Guidelines recommend actively using these tools to guide treatment decisions (31).

### 5.1 Expert opinion

#### 5.1.1 AWS

Along with an accurate and comprehensive medical history, it is important to establish a rapport between the psychiatrist and the patient. In AWS cases, understanding past withdrawal experiences to assess risks of complications like seizures, hallucinations, and DT is essential. **Table 4** lists the key aspects to be enquired about before initiating the treatment for AWS. The next step should be to assess the presenting symptoms. In line with the CIWA-Ar screening tool and observations in clinical practice, mild, moderate, and severe symptoms that typically develop after cessation of alcohol use can be defined (**Supplementary Table 5**). Questionnaires and screening tools such as CIWA-Ar are important for quantifying the severity of withdrawal, awareness of the patient regarding the prognosis of alcohol-related medical problems, as well as tracking the patient’s progress and improvement (**Supplementary Table 4**). CIWA-Ar scale may also help in distinguishing between AWS and anxiety based on certain symptoms like nausea and vomiting, and a combination of tactile, auditory, and visual disturbances observed only with AWS or alcohol use but not with anxiety. Physical and laboratory examinations should also be conducted before deciding on a course of treatment, especially hepatic and renal function, cardiac health, and potential hypokalemia or other electrolyte imbalances.

**Table 4:** Expert opinion on case history, presenting symptoms, and clinical examination for AWS.

Case history	Presenting symptoms	Laboratory examination	Questionnaires/Tools/Manuals
• Socio-demographic details • Pattern of alcohol use (Amount, timing, frequency, place, etc.) • Type of alcoholic beverage used • Duration of use • Features of Alcohol Dependence (craving, tolerance, withdrawal, physical or psychological symptoms, etc.) • Alcohol-related complications (physical, psychological, familial, social, vocational, financial, legal) • Past abstinence attempts • Level of motivation (coming by self or family or on being referred to by another specialist/employer/legal agency) • Medical & psychiatric history • Family history	Red flags/Indicators for serious concerns: • Cognition changes • Hallucinations • Confusion and agitation • Early symptoms like tremors • Repeated withdrawal episodes	**•** Hemogram to assess hyponatremia and hypokalemia among other parameters • Random blood sugar • Liver function tests (serum bilirubin, SGOT, SGPT, GGT) FibroScan Liver (optional) • Renal function test (serum creatinine, blood urea, including electrolytes, absolutely, serum protein, albumin, and certain viral markers) • Any other test required as needed based on the patient’s condition/comorbidities	CIWA-Ar

CIWA-Ar, Clinical Institute Withdrawal Assessment for Alcohol, Revised; GGT, Gamma-glutamyl transferase; SGOT; serum glutamic-oxaloacetic transaminase; SGPT, Serum Glutamic Pyruvic Transaminase.

#### 5.1.2 AD

Most of the factors to be considered for the history of AWS and AD remain common, but there are some unique criteria to be considered for patients with AD (**Table 5**). Typically, the clinical presentation depends upon the amount of alcohol consumed and duration of AD. MSE can be performed for evaluating cognition, psychiatric disorders, acute effects of alcohol, and suicidal risk (**Table 5**). AUDIT and ASSIST scales can be used for screening of unhealthy alcohol use and risk of developing AWS and addressing polysubstance abuse, respectively. DSM-5 criteria and the SADQ for a detailed assessment of physical withdrawal symptoms, psychological dependence, and relief drinking can be used to establish the severity of AD. CAGE questionnaire is strongly recommended in case of high clinical suspicion for patients not disclosing alcohol use (**Table 5**, **Supplementary Table 6**). Among laboratory investigations, complete hemogram and liver function tests are recommended to be performed in resource-constrained settings (**Table 5**). Elevated levels of aspartate aminotransferase (AST), alanine aminotransferase (ALT), gamma-glutamyl transferase (GGT), or bilirubin indicate the severity of AD.

**Table 5:** Expert opinion on case history, presenting symptoms, and clinical examination for AD.

Case history	Presenting symptoms	Laboratory examination	Mental state examination	Questionnaires/Tools/Manuals
• Initiating factors • Most recent incident of consumption •History of addiction or poly-substance use • Ability to control dependence during festivals • Alcohol consumption at work • Availability of alcohol • Complications • History of treatment for alcohol dependence or withdrawal • Childhood history • Family history • Premorbid state • Change in diet • Screening for depression and suicidal thoughts	• Unsteady gait • Difficulty in standing • Slurred speech • Nystagmus • Decreased level of consciousness (e.g., stupor, coma) • Flushed face • Conjunctival infection • Hepatic dysfunction • Pancreatitis • Peripheral Neuropathy • Insomnia • Gastritis • Renal impairment • Increased sweating • Increased alcohol use due to higher tolerance • A strong desire to use alcohol • Difficulty controlling alcohol use • Persistence despite harmful consequences	Same as AWS • Cardiac health • Nutrition status (especially thiamine) • Blood ammonia •Ultrasonography • Amylase • Lipase • Thyroid Stimulating Hormone • Prothrombin time (PT/INR)	1. Cognitive assessment: • Memory problems • Attention & concentration • Judgment Decision making 2. Psychiatric disorder assessment: • Mood disorders • Anxiety • Paranoia • Personality disorders 3. Evaluating the acute effects of alcohol: • Intoxication • Withdrawal: Delirium tremens 4. Evaluating suicidal risk: • Suicidal thoughts or self-harm	• DSM-5 diagnostic criteria • ASSIST • AUDIT • CAGE • SADQ

AWS, alcohol withdrawal syndrome; DSM-5, Diagnostic and Statistical Manual, 5th Edition; AUDIT, Alcohol Use Disorders Identification Test; ASSIST, Alcohol, Smoking and Substance Involvement Screening Test; SADQ, Severity of Alcohol Dependence Questionnaire; PT, prothrombin time; INR, International Normalized Ratio; DT, delirium tremens.

#### 5.1.3 AD with anxiety

Patients with symptoms that are temporarily associated with alcohol use, withdrawal, or intoxication and resolve within weeks of abstinence are more likely to have developed anxiety consequential to alcohol use or dependence. On the contrary, anxiety predating alcohol use or persisting even after abstinence with symptoms independent of alcohol use patterns indicates pre-existing anxiety disorder. A detailed case history, psychiatric evaluation, and observation during abstinence (at least 4–6 weeks) to differentiate the two is recommended, as the course of treatment differs for either condition. Characterizing the severity of anxiety also influences treatment strategies, as mild anxiety may resolve with alcohol cessation and minimal intervention, whereas moderate to severe anxiety often requires intensive and integrated pharmacological treatment to address both conditions (AD and anxiety) simultaneously. Certain symptoms may appear exclusively in this patient profile and should be screened during physical and MSE examination to differentiate from AWS or AD without anxiety (**Table 6**). Differentiating symptoms to identify primary anxiety (preceding alcohol use) or secondary anxiety (due to alcohol use/withdrawal) and cognitive deficit should be checked actively. Identifying triggers (social situations, withdrawal, or specific fears that may drive anxiety and alcohol use) and assessing severity at this stage helps tailor the treatment approaches to individual patient needs. GAD-7 and HAM-A are typically used by physicians and psychiatrists, respectively, in their clinical practice. Both scales can be used to screen for anxiety disorders in patients with AD and to assess their severity (**Table 6**). The laboratory examination stays same as that for AD.

**Table 6:** Expert opinion on case history, presenting symptoms, and clinical examination for AD with anxiety.

Case history	Presenting symptoms	Mental state examination	Questionnaires/Tools/Manuals
• Childhood history • Family history • Medical & psychiatric history • Prescription of mood stabilizers or antipsychotics • Physical, social, psychological, and environmental aspects • Screening for depression and suicidal thoughts • History of addiction or poly-substance use	• Drinking alcohol specifically to alleviate anxiety symptoms • Intense anxiety occurring immediately after alcohol use or during withdrawal, often more severe than in individuals without anxiety • Experiencing obsessive worry about their drinking behavior, its consequences, or their ability to quit • Tremors that worsen in high-stress situations or persist after alcohol withdrawal resolves • Experiencing an excessive fear of alcohol withdrawal symptoms, even before they occur, due to heightened anxiety • Severe sleep problems • Prone to alcohol-induced panic attacks during intoxication, withdrawal, or abstinence	• General appearance and behavior • Psychomotor activity • Speech • Affect • Thought • Perception • Orientation • Attention and concentration • Memory • Intelligence • Abstraction • Judgment • Insight • Level of motivation	• GAD-7 • HAM-A

## 6 Treatment: AWS, AD, and AD with anxiety

Currently, there are no published treatment algorithms delineating the role of physicians and psychiatrists in managing AWS, AD, and AD with anxiety. **Table 7** provides guideline recommendations available for AD, AWS, and co-morbid anxiety. The ASAM and Management of Alcohol Dependence, Ministry of Health and Family Welfare, Government of India, 2017, provides recommendations for the various standard pharmacotherapies and other interventions required for the management of AWS and AD (31, 32). They also offer insights into managing psychiatric co-morbidities such as anxiety with alcohol use (31, 32). Indian clinical practice guidelines and NICE guidelines guide managing generalized anxiety disorder (GAD) (55, 56). A recent expert opinion review offers a treatment algorithm for management of AWS in the Indian clinical practice with recommendations from Indian experts; however, it also highlights the need for separate and clear directives for physicians and psychiatrists (57).

**Table 7:** Guideline recommendations available for AWS, AD, and anxiety (31, 32, 55, 56, 58).

Criteria	Recommendation
Phases	• Initial short- term management phase (detoxification) • Long- term management phase
Inpatient/Outpatient	Based on specific indications
Severity	Validated scales such as CIWA-Ar
Pharmacotherapy	• Severe/Complicated-Benzodiazepines (first line) with front-loading regimen • Mild-to-moderate- Benzodiazepines (first line)/carbamazepine/gabapentin • Mild- Pharmacotherapy or supportive care alone • Long-term management: Acamprosate, disulfiram, naltrexone
Regimen	Fixed-dose, symptom triggered or front loading as per specific clinical scenarios
Psychotherapy	• MET
Supportive care	Nutritional support (multivitamins, thiamine), supportive non-pharmacologic care (family care, environment)
Comorbid anxiety	• Comorbid anxiety: ➢ Pharmacotherapy: SSRIs, SNRI, TCA, mirtazapine, buspirone, venlafaxine, pregabalin, benzodiazepines ➢ Psychotherapy: CBT • Anxiety due to AWS: Alpha2-adrenergic agonists (A2AAs) such as clonidine as an adjunct to benzodiazepine therapy

CBT, cognitive behavior therapy; MET, motivational enhancement therapy; SSRI, selective serotonin reuptake inhibitor; SNRI, serotonin and norepinephrine reuptake inhibitor; TCA, tricyclic antidepressant.

The management protocol for AD usually comprises an initial short-term management phase called ‘detoxification’ and a long-term management phase (31). The ‘detoxification’ phase is also called ‘withdrawal management’ for patients presenting with AWS and aims at reducing the physiological and psychological symptoms associated with withdrawal (32). The decision to treat the patient as an inpatient or an outpatient depends on several factors, including the severity of AD, withdrawal, or symptoms of any comorbid illness, and patient-related criteria such as poor psychosocial support or accessibility to the treatment center (31). The severity of symptoms is recommended to be assessed through validated tools and questionnaires, specific to AWS, AD, and anxiety (**Table 3**) (31, 32). Benzodiazepines are first-line treatment options for severe or complicated AWS and can be administered as fixed-dose, symptom-triggered, or front-loading regimens. The dosing regimens are primarily decided based on the severity of the withdrawal symptoms and the level of clinical monitoring required (31, 32). Pharmacotherapy for mild-to-moderate cases is determined through frequent reassessments and monitoring the need for withdrawal medication. In addition to benzodiazepines, carbamazepine or gabapentin monotherapy can also be considered in patients with moderate withdrawal symptoms, especially if benzodiazepines are contraindicated (32). Patients with mild withdrawal can be managed with pharmacotherapy (carbamazepine or gabapentin) or supportive care alone (32). Once the initial withdrawal management is successful, the Indian guidelines recommend supportive care, which includes managing the nutritional needs of the patients and ensuring an optimal environment with family support to ensure all-around care during recovery (31). Long-term management with acamprosate, naltrexone or disulfiram can prevent relapse and help maintain abstinence (31). Psychotherapy, such as motivational enhancement therapy (MET), delivered in primary care settings, involves principles of empathy, self-efficacy, and optimism and has demonstrated better patient engagement while treating AUDs (31, 32).

For anxiety associated with AWS that is not controlled with benzodiazepines alone, the ASAM guidelines suggest adding alpha2-adrenergic agonists (A2AAs) such as clonidine in the treatment regimen (32). There are no specific recommendations available for managing co-morbid anxiety or anxiety secondary to AD. However, the NICE guidelines suggest treating alcohol misuse first, followed by symptoms of GAD (56). In case anxiety persists after alcohol abstinence, standard pharmacotherapy for GAD (selective serotonin reuptake inhibitor, SSRI; serotonin and norepinephrine reuptake inhibitor, SNRI; tricyclic antidepressant, TCA; mirtazapine; buspirone; venlafaxine; pregabalin; benzodiazepines) can be considered (55, 56, 58). Cognitive behavioral therapy (CBT) can be added as an adjunct to pharmacotherapy in patients who do not respond adequately to the standard treatment. The Indian clinical practice guidelines suggest using benzodiazepines for long-term treatment of anxiety only if other drugs or CBT do not show improvement (55).

### 6.1 Expert opinion

#### 6.1.1 Treatment algorithm for AWS

**Figure 2:**
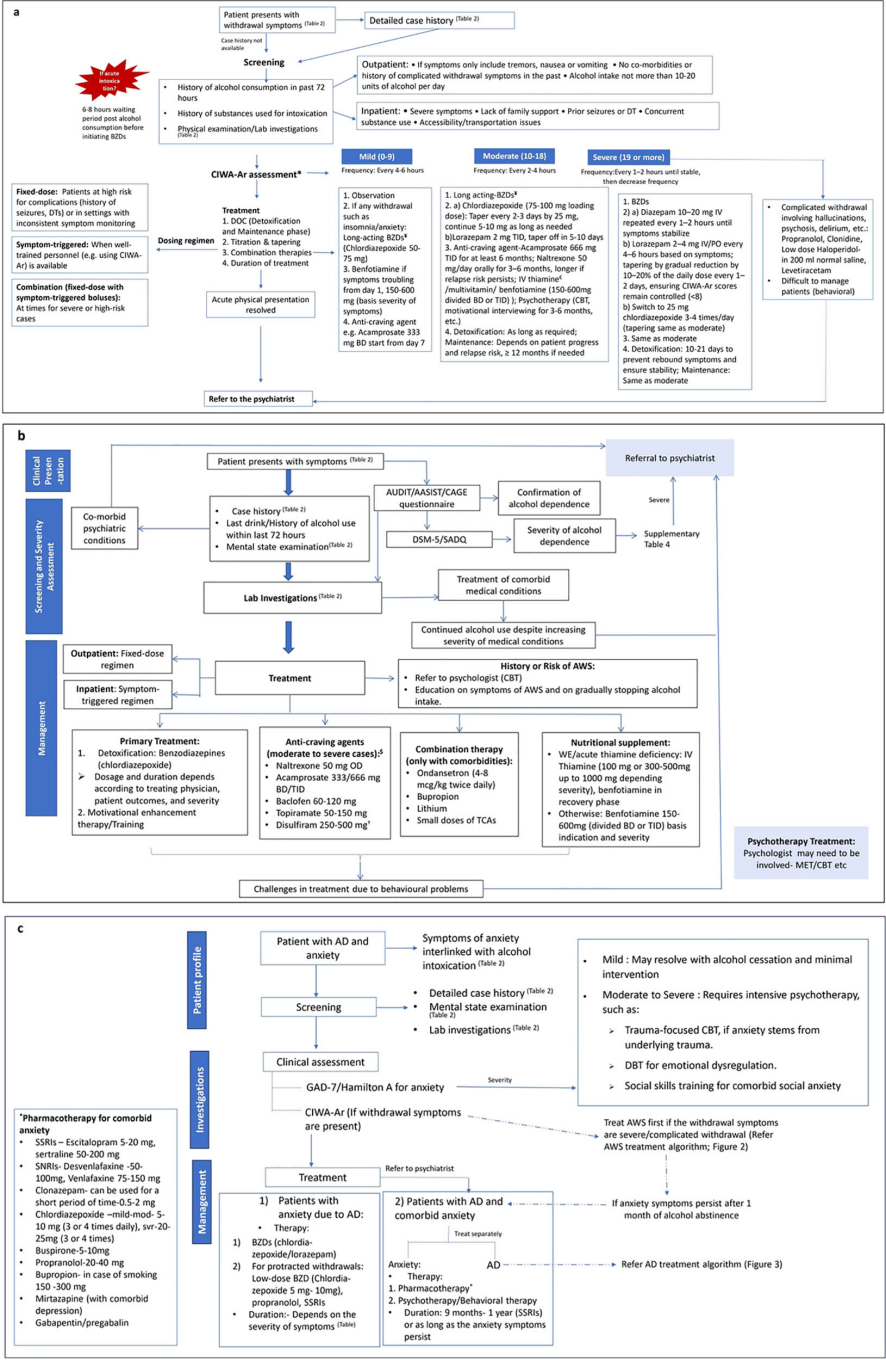
Expert recommended management algorithm for AWS **(A)**, AD **(B)** and AD with anxiety **(C)**. ^*^Document and reassess after medication administration to ensure response ^¥^ Long-acting BZDs are preferred over short-acting BZDs unless the patient is elderly or has impaired liver function or severe agitation ^€^Up to 1000 mg thiamine can be administered if there is risk of Wernicke’s encephalopathy, 1200–1500 mg thiamine in case of Korsakoff’s syndrome ^†^Patient consent should be taken before its administration ^$^Monitor hepatic profile while administering anti-craving agents such as naltrexone. AD, alcohol dependence; AWS, alcohol withdrawal syndrome; BZD, benzodiazepines; BD, twice daily; TID, thrice daily; CBT; cognitive behavior therapy; IV, intravenous; DT, delirium tremens; MET, motivational enhancement therapy; AUDIT, Alcohol Use Disorders Identification Test; ASSIST, Alcohol, Smoking and Substance Involvement Screening Test DSM-5, Diagnostic and Statistical Manual, 5th Edition; SADQ, Severity of Alcohol Dependence Questionnaire; TCA, tricyclic antidepressant; GAD-7, Generalized anxiety disorder 7-item; DBT, Dialectical behavior therapy; SSRI, selective serotonin reuptake inhibitor; SNRI, serotonin and norepinephrine reuptake inhibitor.

The decision regarding inpatient and outpatient treatment is primarily determined by the degree of withdrawal and patient preference but may also be influenced by factors identified during physical examinations and laboratory investigations (**Figure 2A**). Only about 1%–10% of patients with AD/AWS require hospitalization due to the severity of their symptoms. Each of the two dosing regimens, fixed-dose and symptom-triggered, should be preferred in specific scenarios and can even be used in conjunction when necessary (**Figure 2A**). Treatment for AWS typically includes detoxification, titration, and tapering of benzodiazepines, as well as combination therapies, with a duration that may vary, or overlap based on the severity of withdrawal as assessed by CIWA-Ar (**Figure 2A**). Adjunct nutrient supplementation is also often necessary. Thiamine supplementation is recommended because thiamine deficiency is commonly observed in frequent alcohol users. The route of administration depends on severity, with intravenous thiamine recommended as the initial therapy for cases of acute deficiency or Wernicke’s Encephalopathy (WE), and oral thiamine suggested for mild cases (**Figure 2A**). Oral benfotiamine is preferred over oral thiamine due to its better absorption and bioavailability. Vitamin C supplementation is recommended for patients exhibiting symptoms of Korsakoff’s syndrome or experiencing disulfiram-related side effects. Magnesium deficiency and hydration levels should also be monitored and addressed as needed.

Protracted withdrawals may last from 6 to 12 months. Low dose chlordiazepoxide (5 mg) should be administered for a maximum of 2 weeks to ensure a smooth taper and manage residual anxiety in patients experiencing protracted withdrawal.

#### 6.1.2 Treatment algorithm for AD

The treatment algorithms for AD and AWS share similarities (**Figure 2B**). The treatment approaches should be based on severity, as assessed by the standardized questionnaires (**Supplementary Table 7**).

In patients presenting with AD, the detoxification regimen for AWS may be followed as applicable for primary treatment if warranted (**Figure 2A**). MET and training with the help of a psychologist is the other primary treatment modality, especially in the absence of withdrawal or severe AD (**Figure 2B**). Certain patients with mild or moderate AD may benefit from a home-based care setup with the help of family members. Anti-craving drugs can be administered based on the patient’s hepatic profile but are more commonly prescribed for moderate to severe cases with a higher risk of relapse (**Figure 2B**). Thiamine and other nutritional deficiencies can be addressed as elucidated for AWS (**Figure 2B**). Both medical and psychological comorbidities should be appropriately addressed in patients before or concurrent with the treatment for AD (**Figure 2B**). Sleep-related problems can be managed by benzodiazepines, gabapentin, or small doses of TCAs. For patients at risk of developing AWS, education on withdrawal symptoms, gradual cessation of alcohol, and CBT can be helpful.

#### 6.1.3 Treatment algorithm for AD with anxiety

The intensity and type of treatment for AD with anxiety also differ based on the severity of the anxiety disorder (**Figure 2C**).

##### 6.1.3.1 Patients with anxiety due to AD

For treatment of anxiety alone, benzodiazepines are typically administered for a short duration or only when needed, such as during a panic episode. However, benzodiazepines may be required for a longer duration and in a regulated manner in patients with anxiety due to AD. Chlordiazepoxide is well-suited for a longer duration of therapy. A small proportion of patients (2%–3%) may present with inflammatory bowel syndrome (IBS) along with anxiety. Chlordiazepoxide in combination with clidinium bromide may be a suitable therapy in such cases. If anxiety persists after detoxification of AD, then it should be treated as a co-morbid disorder.

##### 6.1.3.2 Patients with AD and comorbid anxiety

Typically recommended to be treated by psychiatrists, comorbid anxiety can be managed by a range of pharmacotherapies (**Figure 2C**), depending on the treating clinician’s preference and patient factors. However, SSRIs remain the treatment of choice for both physicians and psychiatrists for long-term management of comorbid anxiety disorders. Other options, such as SNRIs, may be considered in patients who do not respond to or experience side effects with SSRIs. Sertraline or escitalopram can be safer options in patients with cardiac or renal comorbidities, diabetes, or hypertension.

Many patients with comorbid anxiety also require psychotherapeutic interventions such as CBT and dialectical behavior therapy (DBT). The treatment duration depends on the type of anxiety disorder and may vary accordingly (**Supplementary Table 8**). AD is recommended to be treated as per the treatment algorithm presented in **Figures 2B**, but with careful monitoring of drug-drug interactions between benzodiazepines and anti-anxiety therapy.

### 6.2 Delineating the roles of physicians and psychiatrists

The experts agree on the critical role of physicians in managing alcohol-use related disorders but highlighted the specific scenarios where a physician should refer the patient to a psychiatrist for each of the three indications: For AWS, a) once acute physical problems are resolved, b) presence of complicated withdrawal involving hallucinations, psychosis, DT, etc. c) difficulty in managing patient due to behavioral problems; for AD, a) persistent alcohol consumption despite increasing severity of the medical conditions or b) presence of co-morbid psychiatric disorders; and for AD with anxiety, if the diagnosis for comorbid anxiety is established. Such an integrated treatment plan ensures optimal patient outcomes through a personalized approach.

**Figure 3:**
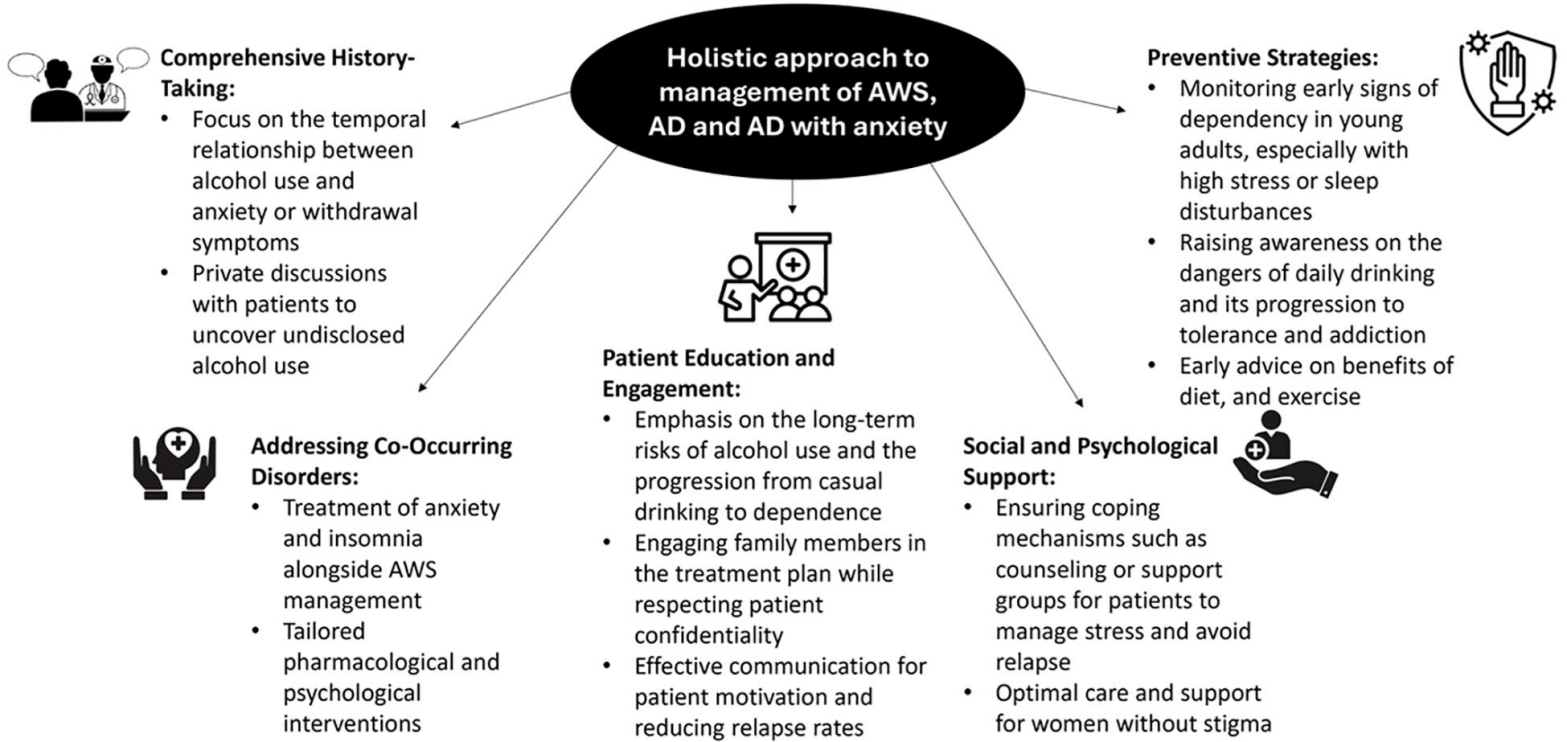
Expert insights on holistic management of AWS, AD, and AD with anxiety.

**Figure 3** illustrates the key recommendations by experts for holistic management of all the three indications.

## 7 Long-acting vs short-acting benzodiazepines in the management of alcohol-related disorders

In AD and AWS, long-acting benzodiazepines are preferred as they can be self-tapered, especially when there is sustained anxiety, active DT or risk of seizures, or usage of front-loading/loading dose regimen (57). Short-acting benzodiazepines are only preferred for episodic anxiety, elderly patients, or patients with significant liver function impairment (57). Short-acting benzodiazepines are associated with a higher risk of adverse effects on abrupt cessation and dependence compared to long-acting benzodiazepines (59). The short half-lives of short-acting benzodiazepines can cause a risk of withdrawal seizures in case of gaps in dosages and pose challenges in discontinuation of the treatment. Such a risk is typically not observed with long-acting benzodiazepines such as chlordiazepoxide and diazepam (60, 61). Short-acting benzodiazepines also carry a risk of misuse/abuse and may ultimately require substitution with a long-acting one (59, 62).

Studies on Indian patients with AWS treated with long-acting benzodiazepines, chlordiazepoxide or lorazepam have demonstrated no definite effect on liver function, especially when used for optimal treatment duration (63, 64). In a retrospective cohort study from an Indian center, the majority of patients with AWS did not have an AST/ALT ratio >2 (86.6%) or AST and GGT >2 times the upper limit of normal (ULN, 87.1%) during post-hospitalization and post-discharge over a six-month study period, indicating that the prescription of the two benzodiazepines was not associated with impaired liver function (64).

### 7.1 Expert opinion

**Figure 4:**
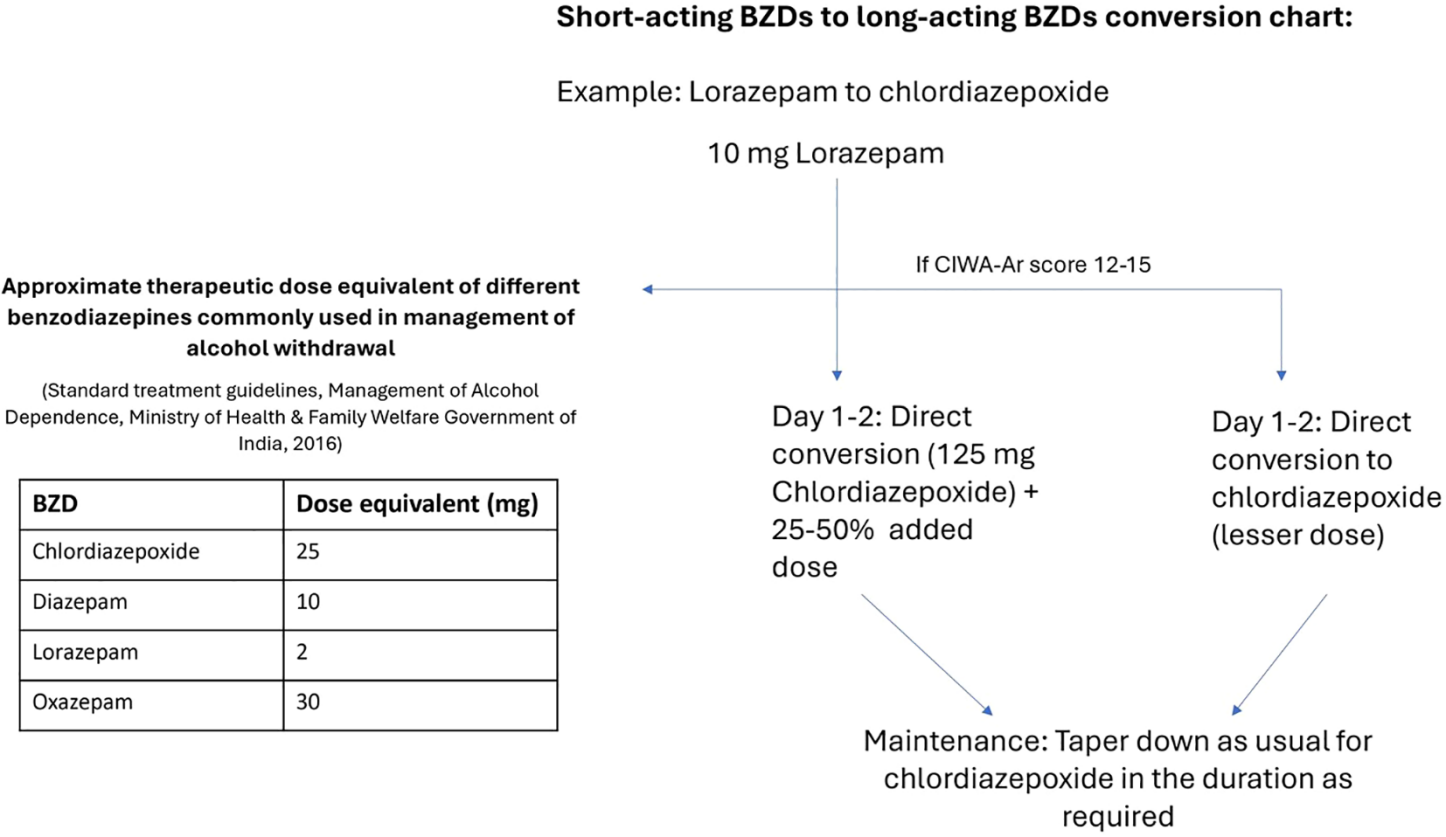
Expert-recommended conversion chart for conversion of short-acting to long-acting benzodiazepines. The example is presented for lorazepam to chlordiazepoxide based on the dose equivalents presented in the table. Dosing frequency, duration, and dosage depend on the regimen used for withdrawal management. BZD, benzodiazepine.

Short-acting benzodiazepines should be administered only in cases with significant liver dysfunction, severe agitation where rapid action is required through intravenous administration, or in elderly patients. However, even in severe cases, short-acting benzodiazepines can be transitioned completely to long-acting benzodiazepines (with appropriate dose equivalents) for maintenance once the patient stabilizes as per the conversion chart presented in **Figure 4**.

Chlordiazepoxide remains the treatment of choice for AD and AWS over other benzodiazepines like lorazepam and oxazepam due to its distinct advantages, such as longer duration of action, fewer adverse effects, smoother withdrawal, and lower abuse potential. Low dose chlordiazepoxide (5 mg) is also effective for tapering off the benzodiazepine treatment and for treating the residual anxiety during protracted withdrawal. Overuse or abuse of chlordiazepoxide is not observed in clinical practice. It is also recommended as a maintenance therapy in alcohol withdrawal owing to its prolonged release action and efficacy in preventing withdrawal symptoms and seizures even after discontinuation.

## 8 Discussion

This expert opinion narrative review provides holistic and comprehensive guidance on the screening, diagnosis, and treatment of patients presenting with AWS, AD, and AD with anxiety. The insights presented in this work will be especially useful for primary care physicians, due to a long-standing gap of clear directives distinguishing their roles from those of psychiatrists, despite often being the first point of contact for patients presenting with alcohol-use-related complications (9, 10).

The burden of AD and AWS continues in India, attributed to starting alcohol consumption at young ages and an increase in social drinking (12, 16). This was corroborated by the available epidemiological studies and the experts’ own clinical experience. The treatment challenges for AUDs in India stem from both physician and patient-related factors (15, 22, 23). However, missed or delayed diagnosis, possibly due to a lack of trained physicians, was identified as a major treatment-related need gap in both the literature and from expert insights. This further highlighted the importance of a systematic checklist of symptoms for diagnosis and diagnosis of exclusion, so that psychiatric comorbidities such as anxiety are not missed while treating AD or AWS.

In line with the published guidelines and clinical evidence, the study emphasizes the use of validated tools (CIWA-Ar, GAD-7, CAGE, AUDIT) that have been extensively studied in clinical settings to screen and assess the severity of AD, AWS, and anxiety (33, 38–54). The recommended pharmacotherapy is also included based on guideline recommendations (31, 32, 55, 56).

The strength of the current work lies in the treatment algorithms, which are based on a combination of recommendations from national and international guidelines and clinical evidence, but incorporate real-world clinical practice insights from the experts. This will enable physicians to implement these management strategies in their day-to-day practice in the Indian scenario. The study attempts to fill an important need—clear and actionable steps for the physicians to diagnose and treat patients presenting with AWS, AD or AD with anxiety, and delineate their roles in the management pathways. Such an attempt at a unified algorithm will help narrow down the variations in management strategies for AUDs, with an overall benefit on the quality of care delivered to the patients.

The major limitation of this work is the potential for bias due to the subjectivity of the expert opinion panel, which may not reflect the practices of all clinicians across diverse Indian geographies. The expert sample distribution also skewed towards psychiatrists, which may have limited the reflection of the perspectives of the physicians. Additionally, expert opinions have the inherent disadvantage of not being well-regulated, though they may prove valuable in the absence of high-quality clinical evidence (65). Lack of a systematic review of literature, which was not feasible given the expanse of the evidence base for the three indications, is also a limitation for the given study.

Despite these limitations, this expert opinion is the first in our knowledge to propose management algorithms for AWS, AD, and AD with anxiety for physicians in India. The next step will be to validate these treatment algorithms in primary care in India to measure their effectiveness and impact on relapse rates in patients with alcohol use. This should be in parallel with the systematic training of physicians to use and apply the recommended screening tools and questionnaires for optimal decision-making. Lastly, there is a need to move beyond acute treatment and focus on preventative measures and integrated care. A multidisciplinary collaboration between primary care providers, psychiatrists, social workers and counsellors can help people with AD before it progresses to more severe pathologies.

## 9 Conclusion

Alcohol use and its associated disorders, such as AWS, AD, and AD with anxiety, continue to pose a significant risk of morbidity and mortality in India. The management of these disorders requires a holistic treatment approach to reduce the burden on both the patients and the caregivers. The detailed directive for physicians and psychiatrists provided in this expert opinion review employs various screening tools and questionnaires to perform a comprehensive assessment and formulate an appropriate treatment strategy using the right drugs and other required psychotherapeutic interventions. Most importantly, the collaboration of physicians with psychiatrists and timely referral will reduce the relapse rates and ensure optimal treatment outcomes.
