# Supplementary material for: Management algorithm for alcohol withdrawal syndrome, alcohol dependence (AD) and AD with anxiety in the Indian population: a narrative review with expert opinion
Source: Front Psychiatry. 2026 Mar 13;17:1758995. doi: 10.3389/fpsyt.2026.1758995 (PMC13021624; doi:10.3389/fpsyt.2026.1758995)
Supplement: Supplementary file 1 [file Supplementaryfile1.docx]

Supplementary Material

Supplementary Table 1. List of participants of the focus group meetings (expert panel)

| **Name** | **Specialty** | **Sex** | **Age** | **Years of Experience** | **Geographic Representation** | **Nature of Practice** | **Zone** |
| --- | --- | --- | --- | --- | --- | --- | --- |
| Dr.H.M. | Psychiatrist | Male | 68 | 44 | Mumbai | Private Practice | West |
| Dr.D. P. | Psychiatrist | Male | 54 | 20 | Mumbai | Private Practice | West |
| Dr.R. G. | Psychiatrist | Male | 70 | 30 | Vashi | Private Practice | West |
| Dr.R. R. D. | Physician | Male | 70 | 35 | Mumbai | Private Practice | West |
| Dr.S. U. | Psychiatrist | Male | 49 | 25 | Thane | Private Practice | West |
| Dr.P. K. | Psychiatrist | Male | 52 | 28 | Solapur | Private Practice | West |
| Dr.S. G. | Psychiatrist | Male | 75 | 52 | Jaipur | Private Practice | North |
| Dr.S.K.T. | Psychiatrist | Male | 70 | 47 | Bhopal | Private Practice | North |
| Dr.S.D. | Physician | Male | 72 | 47 | Bhopal | Private Practice | North |
| Dr.A.P. | Psychiatrist | Male | 46 | 22 | Aurangabad | Private Practice | West |
| Dr.R. S. | Psychiatrist | Male | 41 | 20 | Jaipur | Private Practice | North |
| Dr.A.B. | Physician | Male | 44 | 20 | Pune | Private Practice | West |
| Dr.G.S. | Psychiatrist | Male | 58 | 37 | Barasat | Private Practice | East |
| Dr.S.S. | Psychiatrist | Male | 81 | 55 | Asansol | Private Practice | East |
| Dr.D.B. | Psychiatrist | Male | 38 | 15 | Kolkata | Private Practice | East |
| Dr.U.C. | Psychiatrist | Male | 67 | 45 | Kolkata | Private Practice | East |
| Dr.A.K.D | Psychiatrist | Male | 58 | 36 | Kolkata | Private Practice | East |
| Dr.S.N. | Psychiatrist | Male | 40 | 16 | Kolkata | Private Practice | East |
| Dr.P.P. | Psychiatrist | Male | 73 | 50 | Kolkata | Private Practice | East |
| Dr.A.M. | Psychiatrist | Male | 54 | 31 | Kolkata | Private Practice | East |
| Dr.J.N. | Psychiatrist | Male | 72 | 47 | Kolkata | Private Practice | East |
| Dr.R.H. | Psychiatrist | Male | 49 | 22 | Guwahati | Private Practice | East |
| Dr.D.K.G. | Physician | Male | 68 | 46 | Hoogly | Private Practice | East |
| Dr.T.D. | Physician | Male | 75 | 51 | Hoogly | Private Practice | East |
| Dr.D.R. | Psychiatrist | Male | 55 | 30 | Delhi | Private Practice | North |
| Dr.V.C. | Physician | Male | 50 | 25 | Delhi | Private Practice | North |
| Dr.A.M. | Psychiatrist | Male | 50 | 25 | Chandigarh | Private Practice | North |
| Dr.A.D. | Psychiatrist | Male | 52 | 30 | Noida | Private Practice | North |
| Dr.S.D. | Psychiatrist | Male | 39 | 13 | Ghaziabad | Private Practice | North |
| Dr.S.A. | Psychiatrist | Male | 52 | 28 | Ghaziabad | Private Practice | North |
| Dr.H.S. | Psychiatrist | Male | 50 | 25 | Gurgaon | Private Practice | North |
| Dr.A.K.S. | Physician | Male | 55 | 25 | Ludhiana | Private Practice | North |
| Dr.B.G. | Physician | Male | 67 | 35 | Jalandhar | Private Practice | North |
| Dr.P.C. | Physician | Male | 50 | 23 | Meerut | Private Practice | North |
| Dr.S.M. | Physician | Male | 52 | 25 | Sirsa | Private Practice | North |
| Dr.S.K.T. | Psychiatrist | Male | 69 | 44 | Chennai | Private Practice | South |
| Dr.S.H. | Psychiatrist | Female | 48 | 25 | Mangalore | Private Practice | South |
| Dr.V.S. | Physician | Male | 36 | 11 | Chennai | Private Practice | South |
| Dr.J.S. | Psychiatrist | Female | 40 | 15 | Chennai | Private Practice | South |
| Dr.S.R. | Psychiatrist | Female | 42 | 17 | Chennai | Private Practice | South |
| Dr.V.R. | Psychiatrist | Male | 43 | 19 | Chennai | Private Practice | South |
| Dr.P.T. | Physician | Female | 36 | 12 | Chennai | Private Practice | South |
| Dr.S.K. | Psychiatrist | Male | 40 | 15 | Hyderabad | Private Practice | South |
| Dr.B.S.G.V. | Psychiatrist | Male | 40 | 16 | Warangal | Private Practice | South |
| Dr.T.D. | Physician | Male | 70 | 45 | Kurnool | Private Practice | South |
| Dr.S.M. | Psychiatrist | Male | 44 | 19 | Mysore | Private Practice | South |
| Dr.M.V. | Psychiatrist | Male | 70 | 46 | Ahmedabad | Private Practice | North |
| Dr.S.P. | Psychiatrist | Male | 57 | 32 | Nagpur | Private Practice | West |
| Dr.G.R. | Psychiatrist | Male | 49 | 21 | Hyderabad | Private Practice | South |
| Dr.S.M. | Psychiatrist | Male | 65 | 35 | Delhi | Private Practice | North |
| Dr.R.N. | Psychiatrist | Male | 65 | 35 | Delhi | Private Practice | North |
| Dr.V.I. | Psychiatrist | Male | 47 | 21 | Vijayawada | Private Practice | South |
| Dr.V.S. | Psychiatrist | Male | 37 | 15 | Hyderabad | Private Practice | South |
| Dr.L.G. | Physician | Male | 58 | 34 | Pondicherry | Private Practice | South |

**Supplementary Table 2. Agenda for the focus group meetings**

| **Time Duration** | **Topic** | **Presenter** |
| --- | --- | --- |
| 5 mins | Welcome and Corporate Overview | Abbott |
| 5 mins | Introduction & Context Setting – | Chairperson |
| 10 min | Prevalence and Challenges of AWS, Alcohol dependence & AD with Anxiety | Chairperson & Expert Panel |
| 20 mins | Patient Journey (Diagnosis) in AWS, Alcohol dependence & AD with Anxiety  - Screening (CAGE/ AUDIT Questionnaire)  - Assessment- medical history, physical examination, MSE  -Diagnosis- ICD 11  Referral/ Specialties | Chairperson & Expert Panel |
| 30 mins | Management approach in AWS & Alcohol dependence (IPD/OPD)  -DOC with Regimen  - Duration of Therapy  - Dose Titration (Titration Strategy), Conversion from one benzodiazepine to other  - Relapse Prevention (Maintain Abstinence) | Chairperson & Expert Panel |
| 20 min | Managing multiple relapses/ Complicated cases in Alcohol dependence patients | Chairperson & Expert Panel |
| 30 min | Management approach in Alcohol Dependence with Anxiety patient (IPD/OPD)  -Treatment Algorithm  - Duration of Therapy  - Dose Titration (Titration Strategy), Conversion from one benzodiazepine to other  - Relapse Prevention (Maintain Abstinence) | Chairperson & Expert Panel |
|  | Summary & Closing Remarks | Abbott Medical |
| Abbreviations: AD, alcohol dependence; AUDIT, Alcohol Use Disorders Identification Test; AWS, alcohol withdrawal syndrome; CAGE, Cutting down, Annoyance by criticism, Guilty feeling, and Eye-openers; ICD-11, International Classification of Diseases 11th Revision; IPD, inpatient department; OPD, outpatient department; MSE, mental state examination | | |

**Supplementary Table 3. Comorbidities and SUDs observed with alcohol use-related cases in clinical practice in India**

| **Indication** | **Physicians** | **Psychiatrists** |
| --- | --- | --- |
| **Comorbidities** | | |
| AD | ▪ General: Comorbid anxiety, IBS, insomnia  ▪ 40–60 years: hepatic encephalopathy, behavioral changes, and insomnia | ▪ 40–60 years: Depression, anxiety, amnesia, and cognitive impairment  ▪ >60 years (late onset AD): Age-related chronic insomnia, anxiety, stress, or depression |
| AWS | - | ▪ Behavioral and Psychological Symptoms of Dementia (BPSD)  ▪ Gastric issues or severe hepatic issues  ▪ Heightened anxiety due to unplanned abstinence |
| **SUDs** | | |
| AD | Cannabis (younger patients), opium, nicotine, and morphine | ▪ <20 years of age: Cannabis, binge drinking, and personality disorders (aggressive behavior, excessive gaming, or losing money)  ▪ Females: Smoking |
| **Other observations** | | |
| AD | ▪ Hospitalization in patients >60 years of age typically due to pre-existing medical comorbidities  ▪ Poor nutrition observed frequently | ▪ Alcohol consumption disclosed by only 4%–5% of patients (out of the 40% of patients dependent solely on alcohol)  ▪ Family history of AD noted in adolescents aged 12–14 years |
| AWS | ▪ Often observed in patients treated for AD | ▪ Moderate or severe withdrawal 2–4 days after the patient’s last alcohol consumption observed in most patients  ▪ Complicated withdrawal symptoms including seizure and delirium noted in  20% of this patient subset  ▪ Alcoholic hallucinosis observed in older patients |
| AD with anxiety | ▪ Anxiety and AD overlap observed in approximately 2% of patients, though careful history-taking may reveal a higher prevalence | ▪ Observed in 20%–30% of patients, more frequently than AD alone, and before, during or after the course of AD |
| Abbreviations: AD, alcohol dependence; AWS, alcohol withdrawal syndrome; IBS, inflammatory bowel disease; SUD, substance use disorder | | |

**Supplementary Table 4. The CIWA-Ar scale** (33)

Patient:

Date:

Time (24-hour clock, midnight = 00:00):

Pulse or heart rate, taken for one minute

Blood pressure:

| NAUSEA AND VOMITING —  Ask “Do you feel sick to your stomach? Have you vomited?” Observation.  0 no nausea and no vomiting  1 mild nausea with no vomiting  2  3  4 intermittent nausea with dry heaves  5  6  7 constant nausea, frequent dry heaves and vomiting | TACTILE DISTURBANCES —  Ask “Have you any itching, pins and needles sensations, any burning, any numbness, or do you feel bugs crawling on or under your skin?” Observation.  0 none  1 very mild itching, pins and needles, burning or numbness  2 mild itching, pins and needles, burning or numbness  3 moderate itching, pins and needles, burning or numbness  4 moderately severe hallucinations  5 severe hallucinations  6 extremely severe hallucinations  7 continuous hallucinations |
| --- | --- |
| TREMOR —  Arms extended and fingers spread apart. Observation.  0 no tremor  1 not visible, but can be felt fingertip to fingertip  2  3  4 moderate, with patient's arms extended  5  6  7 severe, even with arms not extended | AUDITORY DISTURBANCES —  Ask “Are you more aware of sounds around you? Are they harsh? Do they frighten you? Are you hearing anything that is disturbing to you? Are you hearing things you know are not there?” Observation.  0 not present  1 very mild harshness or ability to frighten  2 mild harshness or ability to frighten  3 moderate harshness or ability to frighten  4 moderately severe hallucinations  5 severe hallucinations  6 extremely severe hallucinations  7 continuous hallucinations |
| PAROXYSMAL SWEATS — Observation.  0 no sweat visible  1 barely perceptible sweating, palms moist 2  3  4 beads of sweat obvious on forehead  5  6  7 drenching sweats | VISUAL DISTURBANCES —  Ask “Does the light appear to be too bright? Is its color different? Does it hurt your eyes? Are you seeing anything that is disturbing to you? Are you seeing things you know are not there?” Observation.  0 not present  1 very mild sensitivity  2 mild sensitivity  3 moderate sensitivity  4 moderately severe hallucinations  5 severe hallucinations  6 extremely severe hallucinations  7 continuous hallucinations |
| ANXIETY —  Ask “Do you feel nervous?” Observation.  0 no anxiety, at ease  1 mild anxious  2  3  4 moderately anxious, or guarded, so anxiety is inferred  5  6  7 equivalent to acute panic states as seen in severe delirium or acute schizophrenic reactions | HEADACHE, FULLNESS IN HEAD —  Ask “Does your head feel different? Does it feel like there is a band around your head?” Do not rate for dizziness or lightheadedness. Otherwise, rate severity.  0 no present  1 very mild  2 mild  3 moderate  4 moderately severe  5 severe  6 very severe  7 extremely severe |
| AGITATION —  Observation.  0 normal activity  1 somewhat more than normal activity  2  3  4 moderately fidgety and restless  5  6  7 paces back and forth during most of the interview, or constantly thrashes about | ORIENTATION AND CLOUDING OF SENSORIUM —  Ask “What day is this? Where are you? Who am I?”  0 oriented and can do serial additions  1 cannot do serial additions or is uncertain about date  2 disoriented for date by no more than 2 calendar days  3 disoriented for date by more than 2 calendar days  4 disoriented for place/or person |

Total CIWA-A Score:

Rater's Initials:

Maximum Possible Score 67

**Supplementary Table 5. Mild, moderate, severe, and atypical withdrawal symptoms encountered in patients with AWS**

| **Mild symptoms** (most common)  (6-12 hours after alcohol reduction/cessation) | **Moderate symptoms** (12-24 hours after alcohol reduction/cessation) | **Severe symptoms** (less common, but serious)  (48-96 hours after alcohol reduction/cessation) | **Atypical withdrawal symptoms** |
| --- | --- | --- | --- |
| - Tremors (shaking) - Anxiety or irritability - Insomnia or restlessness - Headache - Palpitations - Diaphoresis - Gastrointestinal upset (nausea/vomiting) - Mild tachycardia and elevated blood pressure | - Agitation - Hallucinations (visual, auditory, or tactile) without alcoholic hallucinosis - Marked anxiety - Worsening tremors | - Seizures (generalized tonic-clonic; often single or in clusters) - Delirium Tremens (DTs) (confusion, hallucinations, severe autonomic hyperactivity like tachycardia, hypertension, hyperthermia) - Severe agitation | - Protracted Withdrawal Syndrome (Lasting weeks to months, dysphoria or depression, fatigue, persistent anxiety or irritability and Insomnia - Isolated withdrawal seizures - Psychiatric disorder-like presentations: Paranoia, catatonia-like behavior, misidentification syndrome - Autonomic instability without overt tremens |

**Supplementary Table 6: CAGE questionnaire** (48)

| **CAGE questionnaire** |
| --- |
| Have you ever felt you should **C**ut down on your drinking? |
| Have people **A**nnoyed you by criticizing your drinking? |
| Have you ever felt bad or **G**uilty about your drinking? |
| Have you ever had a drink first thing in the morning to steady your nerves or to get rid of a hangover (**E**ye opener) |

**Scoring:** Item responses on the CAGE are scored 0 or 1, with a higher score an indication of alcohol problems. A total score of 2 or greater is considered clinically significant.

**Supplementary Table 7.** **Treatment approaches for AD based on severity**

| **Treatment parameter** | **Mild AD** | **Moderate AD** | **Severe AD** |
| --- | --- | --- | --- |
| Setting | Outpatient | Structured outpatient | Inpatient or residential |
| Withdrawal Management | Rarely required | May require outpatient detoxification | Always require supervised detoxification |
| Medication Use | Optional | Common | Essential |
| Therapy Focus | Brief interventions, CBT | IOP, CBT, family therapy | Intensive therapy, relapse prevention |
| Support | Self-help groups | Peer groups and structured follow-up | Long-term care and aftercare programs |
| Abbreviations: AD, alcohol dependence; CBT, cognitive behavior therapy; IOP, intensive outpatient program | | | |

**Supplementary Table 8. Treatment duration for patients with anxiety with AD**

| **Type of Anxiety** | **Initial Phase** | **Maintenance** | **Total Duration** |
| --- | --- | --- | --- |
| Alcohol-Induced Anxiety | 4–12 weeks | Not typically needed | ~3 months |
| Primary Anxiety Disorder | 6–12 months | 6–12 months or longer | 1–2 years or more |
| Persistent Anxiety in Abstinence | 6–12 months | As needed | ~6–12 months or longer |
